# The kinase Bud32 regulates iron homeostasis in fungal pathogen *Cryptococcus neoformans*

**DOI:** 10.3389/fimmu.2025.1624237

**Published:** 2025-07-25

**Authors:** Yuanyuan Ma, Bo Pan, Wenzhi Lei, Wenjie Fang, Weihua Pan, Wanqing Liao, Bin Xu, Peng Xue

**Affiliations:** ^1^ Nantong Key Laboratory of Environmental Toxicology, Department of Occupational Medicine and Environmental Toxicology, School of Public Health, Nantong University, Nantong, China; ^2^ Department of Dermatology, Shanghai Key Laboratory of Molecular Medical Mycology, Second Affiliated Hospital of Naval Medical University, Shanghai, China; ^3^ Jiangxi Key Laboratory of Oncology (2024SSY06041), JXHC Key Laboratory of Tumor Metastasis, Jiangxi Cancer Hospital, The Second Affiliated Hospital of Nanchang Medical College, Nanchang, China

**Keywords:** cryptococcosis, Bud32, iron homeostasis, proteome, metabolomics, phosphoproteomics

## Abstract

**Introduction:**

The ability to acquire iron and maintain iron homeostasis is crucial for the virulence of the human pathogenic fungus *Cryptococcus neoformans*. This study investigates the role of Bud32, a core virulence kinase and component of the KEOPS complex, within the iron regulatory network of *C. neoformans.*

**Methods:**

We used gene deletion techniques to study the phenotypic effects of *BUD32* gene knockout and conducted proteomic and metabolomic analyses to assess changes in protein expression and metabolite levels in the mutant. Additionally, we performed *in vivo* phosphoproteomics analysis to evaluate Bud32 impact on iron regulatory proteins.

**Results:**

Our findings revealed that deletion of *BUD32* gene significantly impaired growth in iron-limiting environments, leading to notable alterations in the expression of iron transport and iron-sulfur cluster (ISC)-containing proteins. Specifically, Bud32 was shown to modulate ISC assembly and influence the activity of key iron-sulfur binding proteins, including Grx4, Cir1, and HapX. Metabolic profiling indicated changes in 696 metabolites, with reductions in biliverdin levels. Additionally, *BUD32* gene deletion resulted in widespread changes in the phosphorylation status of numerous proteins, including the iron regulators Cir1 and Rim101.

**Conclusion:**

These findings provide evidence for the involvement of the kinase Bud32 in regulating iron homeostasis in *C. neoformans*, thereby contributing to our understanding of its virulence mechanisms.

## 1 Introduction

The incidence of invasive fungal diseases is experiencing a marked increase. This trend poses significant treatment challenges, stemming from an incomplete understanding of their pathogenic mechanisms and a limited repertoire of antifungal agents (1–3). Current research is concentrating on the development of more effective antifungal drugs, the exploration of vaccine candidates, and a deeper understanding of the pathogenic mechanisms employed by fungi (4–6). According to data from the World Health Organization (WHO), the four critical priority fungal pathogens for humans are *Cryptococcus neoformans*, *Candida auris*, *Aspergillus fumigatus*, and *Candida albicans* (7). In immunocompromised hosts, such as patients with HIV/AIDS and organ transplant recipients, cryptococcal meningitis is one of the most common invasive fungal diseases, with the majority of cases caused by *C. neoformans* (8). It is estimated that globally, approximately 180,000 deaths related to AIDS each year are caused by cryptococcal meningitis, accounting for 15% of AIDS-related fatalities (9, 10). Currently, the clinical treatment options for cryptococcosis include amphotericin B (which has high toxicity), flucytosine, and triazole antifungal drugs. However, treatment failures and recurrent infections during the management of cryptococcal meningitis are common. In recent years, the widespread and prolonged use of fluconazole has led to a gradual increase in resistance of *C. neoformans* to this class of drugs, further elevating the risk of clinical treatment failure for patients (10–12).

The amount of free iron in the host is extremely limited, approximately 10–^18^ M. Proteins such as lactoferrin and transferrin bind iron (Fe^3+^) with high affinity, effectively sequestering it and potentially hindering its utilization by pathogens (13, 14). To proliferate and initiate disease, pathogenic microorganisms must overcome this host iron restriction and acquire approximately 10–^6^ to 10–^7^ M of iron (15–18). *C. neoformans* can hijack the host iron through iron acquisition systems to sustain its proliferation and the formation of virulence factors (15, 19–21). Excess iron in liver transplant patients is considered a significant factor contributing to the high incidence of cryptococcosis and other fungal infections (22–24). Similarly, excessive iron in HIV/AIDS patients is also regarded as an important factor for the high prevalence of cryptococcal disease (25, 26). Overall, there is a close relationship between iron and fungal infections, highlighting the urgent need for in-depth research on the pathogen sensing, uptake, and homeostatic regulation of iron, which could provide a basis for effective treatment of fungal infections.

Iron uptake functions serve as drug targets, potential drug delivery systems, and vaccine candidates (27–30). The iron uptake system has garnered attention as a therapeutic target against clinical pathogens. Research has shown that combining iron chelators with antibiotics and other drugs has been widely explored for treating bacterial and parasitic infections and is considered a potential strategy for anticancer chemotherapy (28, 31, 32). Furthermore, toxic analogs of heme, such as non-iron metalloporphyrins (e.g., gallium porphyrin IX), exhibit therapeutic potential by inhibiting the growth of bacterial and fungal pathogens through dependence on heme uptake systems (33–35). Studies have also identified the emergence of drugs affecting iron uptake, further validating the potential for treating fungal diseases. For example, chloroquine can accumulate in macrophage phagosomes and neutralize the acidic pH of this organelle, thereby inhibiting the growth of various intracellular pathogens, including *C. neoformans* and *Histoplasma capsulatum* (36, 37). Additionally, lactoferrin, an important iron-chelating agent, has been extensively studied for its role in combating parasitic infections (38, 39). Furthermore, extracellular proteins responsible for iron uptake in bacterial and fungal pathogens are also considered potential vaccine candidates (40, 41).

The core of iron homeostasis regulation in *C. neoformans* is the iron regulatory factor Cir1 (*Cryptococcus* iron regulator 1), which can sense the availability of iron and regulate three classic virulence factors of *C. neoformans* (melanin synthesis, polysaccharide capsule synthesis, and growth at the host body temperature 37°C) (42). During infection, *C. neoformans* uses the GATA transcription factor Cir1 to sense the availability of iron and regulate iron uptake systems, as well as the intracellular balance of iron, ensuring successful competition for iron resources to support its proliferation within the host. Cir1 regulates all iron uptake systems in *C. neoformans*, including the high-affinity iron uptake system. High-affinity iron uptake [mediated by the iron oxidase Cfo1 (*Cryptococcus* ferroxidase 1) and the iron transporter Cft1 (*Cryptococcus* ferric transporter 1)] is essential for fungal virulence expression and proliferation in the central nervous system (43, 44). The iron acquisition system of *C. neoformans* includes high-affinity ferrous iron uptake, iron chelator transport proteins, and iron extraction from heme or hemoglobin (21). The high-affinity iron uptake system first reduces ferric iron to ferrous iron using iron reductases (Fre2 (Ferric reductase transmembrane component 2) and Fre4 (Ferric-chelate reductase 4)). Subsequently, the copper-dependent iron oxidase Cfo1 oxidizes ferrous iron, while the coupled iron transporter Cft1 transports ferric iron into the cell. *C. neoformans* utilizes the high-affinity iron uptake system to acquire iron from transferrin and the major iron storage protein ferritin. Although *C. neoformans* lacks the ability to synthesize its own iron chelators, it can obtain ferric iron by utilizing chelators produced by other microorganisms. Additionally, *C. neoformans* can extract iron from heme, which is the richest source of iron in the vertebrate host. The heme transporter protein Cig1 (Cytokine inducing-glycoprotein) (34) plays a role in heme utilization, along with associated processes such as clathrin-mediated endocytosis, the endosomal sorting complex required for transport (ESCRT) pathway, and other endomembrane transport functions (21). All of the above iron uptake systems are regulated by Cir1, so elucidating the Cir1-mediated iron homeostasis regulatory network could provide strong theoretical support for understanding the mechanisms by which *C. neoformans* produces virulence factors. GlutaRedoXin 4 (Grx4) is a binding partner of the iron regulatory factor Cir1 and is involved in the regulation of iron homeostasis in *C. neoformans* (45). Mutations in the GRX domain of Grx4 lead to defects in melanin synthesis (45). HapX is also involved in regulating iron homeostasis in *C. neoformans*, including iron-dependent functions under iron-limited conditions, such as mitochondrial gene expression. RNA-Seq and ChIP-Seq experiments indicate that Cir1 directly regulates the expression of the *HAPX* gene, with Cir1 suppressing *HAPX* expression under high iron conditions (46, 47). The pH-responsive regulator Rim101 also regulates iron uptake functions including Cig1 and plays a major role in cell wall remodeling (48–50).

The KEOPS (kinase, putative endopeptidase, and other proteins of small size) complex in *C. neoformans* exhibits a conserved linear arrangement of Pcc1-Kae1-Bud32-Cgi121 (51). This arrangement serves critical evolutionary functions, including growth, stress responses, sexual development, and tRNA modification, while also fulfilling unique roles in regulating the production of virulence factors and overall pathogenicity (51). A distinct characteristic of Bud32 is its unique extended loop structure, which is essential for the KEOPS complex functionality in *C. neoformans*. Bud32 is involved in the regulation of key virulence factors, such as capsule formation and melanin production, and is essential for mouse infection (51, 52). Our study reveals that Bud32, a core virulence kinase, plays a significant role in maintaining iron homeostasis in *C. neoformans*. Deletion of *BUD32* gene disrupts this balance, resulting in altered expression of proteins involved in iron regulation and a notable decrease in metabolite levels, particularly biliverdin. Additionally, Bud32 influences the assembly of iron-sulfur clusters and the phosphorylation of iron regulatory factors, such as Cir1 and Rim101. Collectively, these findings underscore the potential role of Bud32 in regulating iron homeostasis within *C. neoformans* and highlight its importance in the virulence.

## 2 Materials and methods

### 2.1 Strain and media

This study employed strains: the wild-type (WT) *Cryptococcus neoformans* var. *grubii* H99 and the deletion mutants *bud32*Δ. The primers used for constructing the *bud32*Δ mutant are provided in **Supplementary Table S1**. The Bud32 homolog gene sequence (CNAG_02712) was obtained from the *C. neoformans* var. *grubii* serotype A genome database (https://www.broadinstitute.org/fungal-genome-initiative/cryptococcus-neoformansserotype-genome-project). To construct the mutant *bud32*Δ, we replaced the entire open reading frame (870 base pairs) of the BUD32 gene with a deletion cassette using homologous recombination. This cassette was amplified using specific primers (Bud32 up_F, Bud32 up_R, Bud32 (nourseothricin-resistance gene NAT)_F, Bud32 (nourseothricin-resistance gene NAT)_R, Bud32 down_F, and Bud32 down_R) and introduced into the wild-type strain through biolistic transformation, following the previous protocols (53–55). Positive transformants were confirmed through PCR. To investigate the alterations in proteomics, metabolomics, and phosphoproteomics between the WT and the *bud32*Δ mutant strains, cells were cultured in YPD medium for approximately 16 hours at 30°C. The cultures were then harvested, washed twice with PBS, and the resulting pellets were frozen in liquid nitrogen before being stored at -80°C. Iron-limiting media were created using defined low-iron medium (LIM) and yeast nitrogen base (YNB with amino acids adjusted to pH 7.0) supplemented with 150 μM bathophenanthroline disulfonate (BPS), as previously detailed in the literature (19, 43, 56).

### 2.2 Capsule formation and melanin production

Capsule formation was analyzed under differential interference contrast (DIC) microscopy after a 16-hour incubation at 30°C in LIM and subsequent staining with India ink. Meanwhile, melanin production was investigated using L-3,4-dihydroxyphenylalanine (L-DOPA) plates supplemented with 0.1% glucose.

### 2.3 Serial spot dilution assays

Overnight fungal cultures were washed twice with PBS and adjusted to 2 x 10^7^ cells/ml. Tenfold serial dilutions were made, and 5 μl of each dilution (ranging from 10^5^ to 10^0^ cells) was spotted onto agar plates. The plates were incubated for 2 days at 30°C or 37°C, followed by photography.

### 2.4 4D-FastDIA quantitative proteome studies

Cell pellets were ground in liquid nitrogen and subsequently subjected to sonication in a lysis buffer. This lysis buffer contained 1% Triton X-100, 1% protease inhibitor cocktail, 10 mM dithiothreitol, 50 μM PR-619, 50 mM NAM, 2 mM EDTA, 3 μM TSA, and 1% phosphatase inhibitor for the purpose of preventing phosphorylation. After adding Tris-saturated phenol and vortexing, proteins were precipitated with ammonium sulfate in methanol, washed, and reconstituted in 8 M urea. Concentration was measured using the BCA protein assay Kit (Beyotime, Cat. No. P0011). Samples were precipitated with 20% TCA and incubated at 4°C. After centrifugation, proteins were washed with acetone and dissolved in 200 mM TEAB. Trypsin was added (1:50 ratio) for overnight digestion at 37°C, followed by reduction with DTT and alkylation with iodoacetamide. Peptides were purified using a Strata X column. Tryptic peptides were dissolved in solvent A, which consisted of 0.1% formic acid and 2% acetonitrile in water, and loaded onto a reversed-phase column. A gradient elution was performed with solvents A and B, with solvent B containing 0.1% formic acid and 90% acetonitrile in water. Peptides were analyzed using an Orbitrap Exploris 480 mass spectrometer, with MS and MS/MS scans at specified resolutions and collision energies. Data-independent acquisition (DIA) data were analyzed using Spectraunt, matching spectra against a *C. neoformans* database. Trypsin/P cleavage was selected, with fixed modifications and FDR under 1%. Differentially expressed proteins underwent functional enrichment analysis via Fisher’s exact test, retaining terms with fold enrichment >1.5 or <0.67 and p-value <0.05. GO analysis is a bioinformatics method for annotating and classifying proteins based on their functions (57). It uses software like eggnog-mapper to extract GO IDs from proteins, facilitating classification into biological processes, cellular components, and molecular functions for comprehensive insights in proteomics projects. The R packages GSEABase (58) and GOstats (59) were employed to carry out the enrichment analyses, while the ggplot2 package (60) was utilized to visualize the results of these enrichment tests.

### 2.5 Metabolomics studies

Cell pellets were thawed and homogenized, then extracted with a methanol-water solution. After three freeze-thaw cycles and centrifugation, the supernatant was collected, stored, and then transferred for LC-MS analysis. Samples were analyzed via LC-MS using a T3 column. Positive ion mode used a gradient of 0.1% formic acid in water and acetonitrile, with a flow rate of 0.4 mL/min and injection volume of 4 μL. Negative ion mode followed the same gradient. Data acquisition utilized Analyst TF 1.7.1 in IDA mode, with specific source parameters for positive and negative modes. PCA was performed using R prcomp function after scaling data for unit variance to ensure accurate analysis. Differential metabolites were identified using VIP (VIP > 1) and *P*-value (< 0.05, Student’s t-test) from OPLS-DA results. Data underwent log transformation (log_2_) and mean centering, with a permutation test of 200 iterations to validate the model and prevent overfitting. Metabolites were annotated with the KEGG Compound database and subsequently mapped to the KEGG Pathway database. Pathways with significant enrichment were identified using a hypergeometric test to calculate P-values for the specified metabolite list.

### 2.6 Phosphoproteomics studies

Samples were ground in liquid nitrogen and sonicated in lysis buffer. Equal volumes of Tris-saturated phenol were added, and the mixture was vortexed and centrifuged. Proteins were precipitated with methanol, washed, and redissolved in 8 M urea for concentration determination using a BCA kit. The sample was treated with 20% TCA for protein precipitation, followed by centrifugation and acetone washing. The protein was redissolved in 200 mM TEAB, digested with trypsin overnight, reduced with dithiothreitol, alkylated with iodoacetamide, and desalted using a Strata X SPE column. Peptide mixtures were incubated with IMAC microspheres in loading buffer. After washing to remove non-specific peptides, phosphopeptides were eluted with 10% NH_4_OH. The supernatant containing phosphopeptides was collected and lyophilized for subsequent LC-MS/MS analysis. Tryptic peptides were loaded onto a reversed-phase column and separated using a gradient on an EASY-nLC 1200 UPLC system. The Orbitrap Exploris 480 analyzed the peptides. Spectral libraries were built using Spectronaut, with modifications specified and an FDR < 1% for database searching against the *C. neoformans* dataset.

## 3 Results

### 3.1 Bud32 is required for major virulence factor formation

Previous studies have indicated that Bud32 is important for producing virulence factors (51, 52). Here, we deleted the *BUD32* gene, following methods similar to those previously reported. Specifically, we investigated the role of Bud32 in the production of key virulence factors in *C. neoformans*, focusing on capsule formation, melanin production, and growth at 37°C. We first assessed capsule formation in both the WT strain and three independent *bud32*Δ mutants (*bud32*Δ#1, *bud32*Δ#2, *bud32*Δ#3). We used low-iron medium as our capsule induction medium, whereas Littman and FBS (10% fetal bovine serum and 90% PBS) media were employed in their studies. The results were obtained through India ink staining, which allows for the visualization of the capsule surrounding the yeast cells. As shown in **Supplementary Figure S1A, B**, the WT strain exhibited a robust capsule, characterized by a clear halo around the cells after India ink staining, indicating successful capsule formation. In stark contrast, the *bud32*Δ mutants displayed a significantly reduced capsule, confirming that Bud32 is essential for the production of this critical virulence factor. To evaluate melanin production, we performed spot assays on L-DOPA plates for each strain. The WT strain demonstrated substantial melanin production, indicated by dark pigmentation on the plates. However, the *bud32*Δ mutants showed a markedly diminished melanin production capacity, which was visually evident as a lack of dark coloration compared to the WT (**Supplementary Figure S1C**). This finding underscores the potential role of Bud32 in regulating melanin biosynthesis, which is associated with the virulence of *C. neoformans*. Next, we examined the sensitivity of each strain to temperature variations, particularly at 30°C and 37°C, by conducting spot assays on YPD medium. The WT strain exhibited robust growth at both temperatures, indicating its adaptability. Conversely, the *bud32*Δ mutants displayed compromised growth at 37°C, suggesting that Bud32 is important for maintaining cellular functions and viability under elevated temperatures commonly encountered in the host environment (**Supplementary Figure S1D**). Overall, Bud32 is required for the formation of the major virulence factors in *C. neoformans*, including capsule, melanin and growth at host body temperature 37°C.

### 3.2 Bud32 is essential for growth under iron limiting conditions

**Figure 1:**
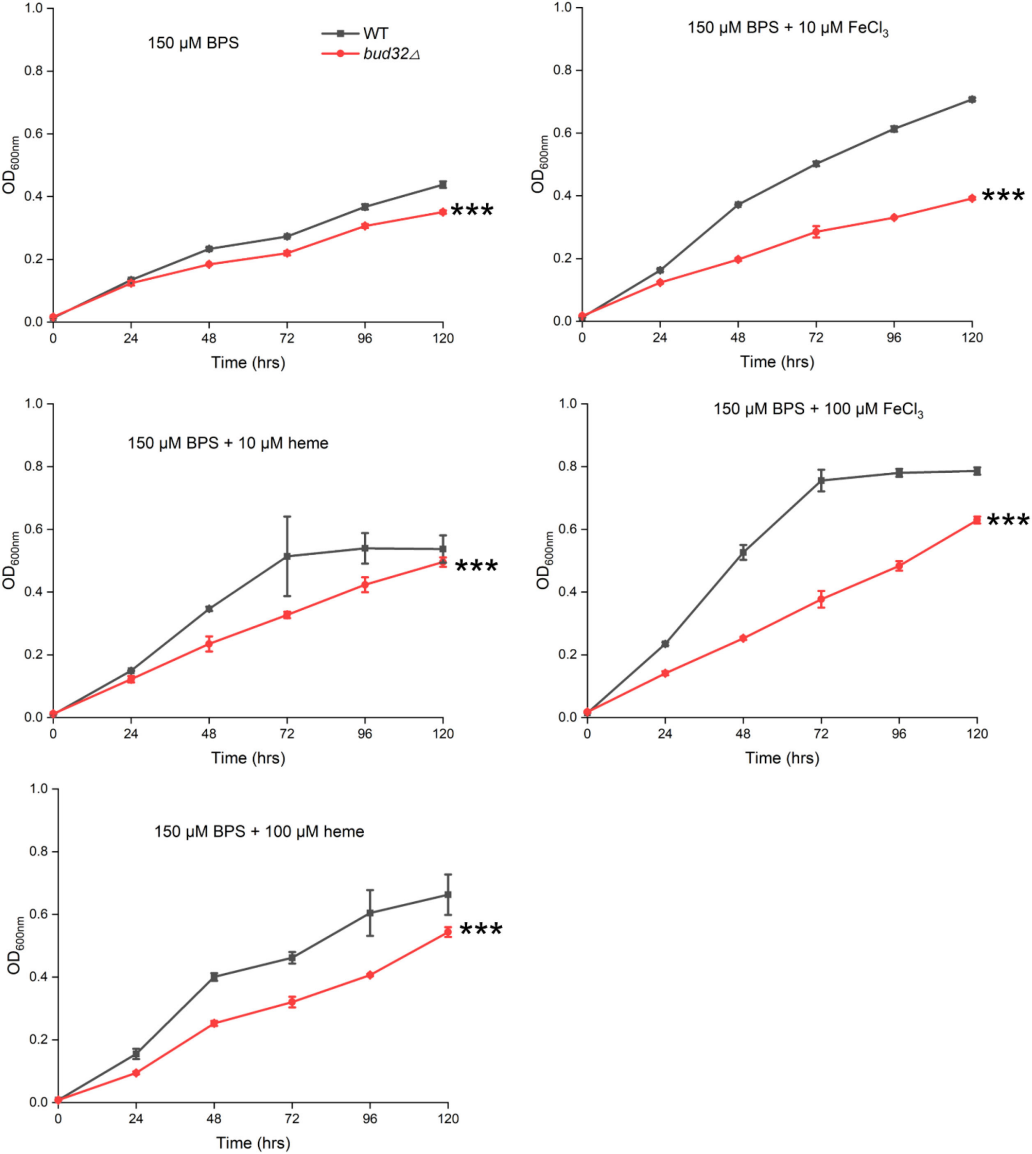
The role of Bud32 for optimal growth with inorganic iron or heme as the sole iron source. WT cells or the *bud32*Δ mutant were grown in liquid YNB medium supplemented with 150 mM BPS, with or without the addition of FeCl_3_ or heme as the iron source. The cultures were then incubated at 30°C, and the optical density at 600 nm (OD_600_) was measured. Mean values of three biological replicates are shown ± standard deviation (SD). *** (two-way ANOVA, *P*<0.001).

We then tested whether Bud32 is involved in iron utilization by growing the WT and *bud32*Δ mutant strains under the low-iron conditions. We utilized liquid YNB medium supplemented with 150 mM BPS to chelate iron, simulating low-iron environments. We further tested the ability of the *bud32*Δ mutant to utilize alternative iron sources, specifically FeCl_3_ and heme. The fungal cultures were incubated at 30°C, and optical density (OD_600_) measurements were taken to evaluate growth. As illustrated in **Figure 1**, the WT cells demonstrated optimal growth in the presence of either FeCl_3_ or heme as iron sources, as evidenced by higher OD_600_ readings. In contrast, the *bud32*Δ mutant exhibited significantly impaired growth when these iron sources were provided, indicating its inability to effectively utilize inorganic iron or heme (**Figure 1**). These results suggest that Bud32 is important for the optimal growth of *C. neoformans* in environments where iron availability is limited.

### 3.3 Impact of Bud32 on protein and metabolite levels related to iron homeostasis and metabolism

**Figure 2:**
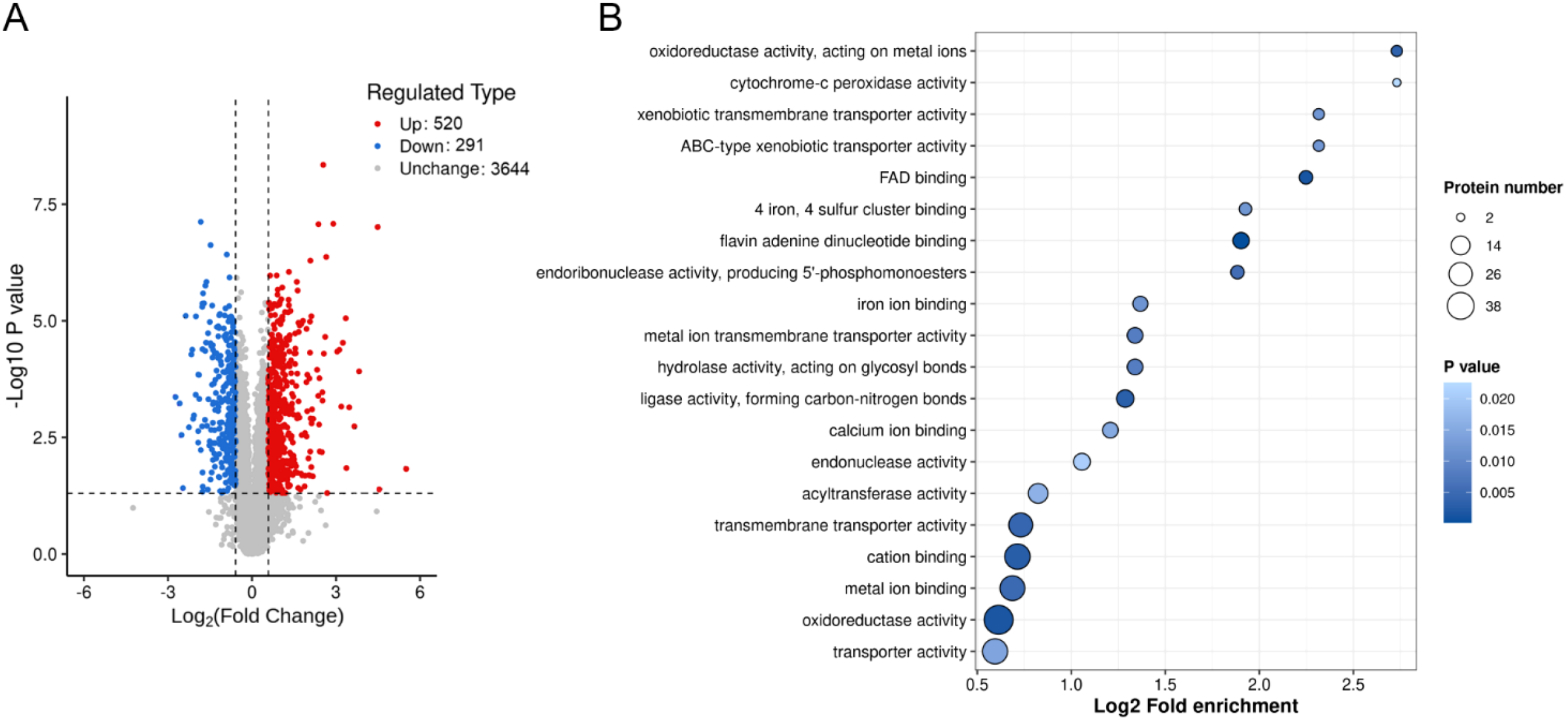
The effects of Bud32 on protein levels related to iron ion binding and iron sulfur cluster binding. **(A)** Deletion of *BUD32* gene resulted in 520 up-regulated proteins (in red) and 291 down-regulated proteins (in blue). **(B)** GO molecular function analysis for the proteomic comparison between WT control and *bud32*Δ mutant.

**Figure 3:**
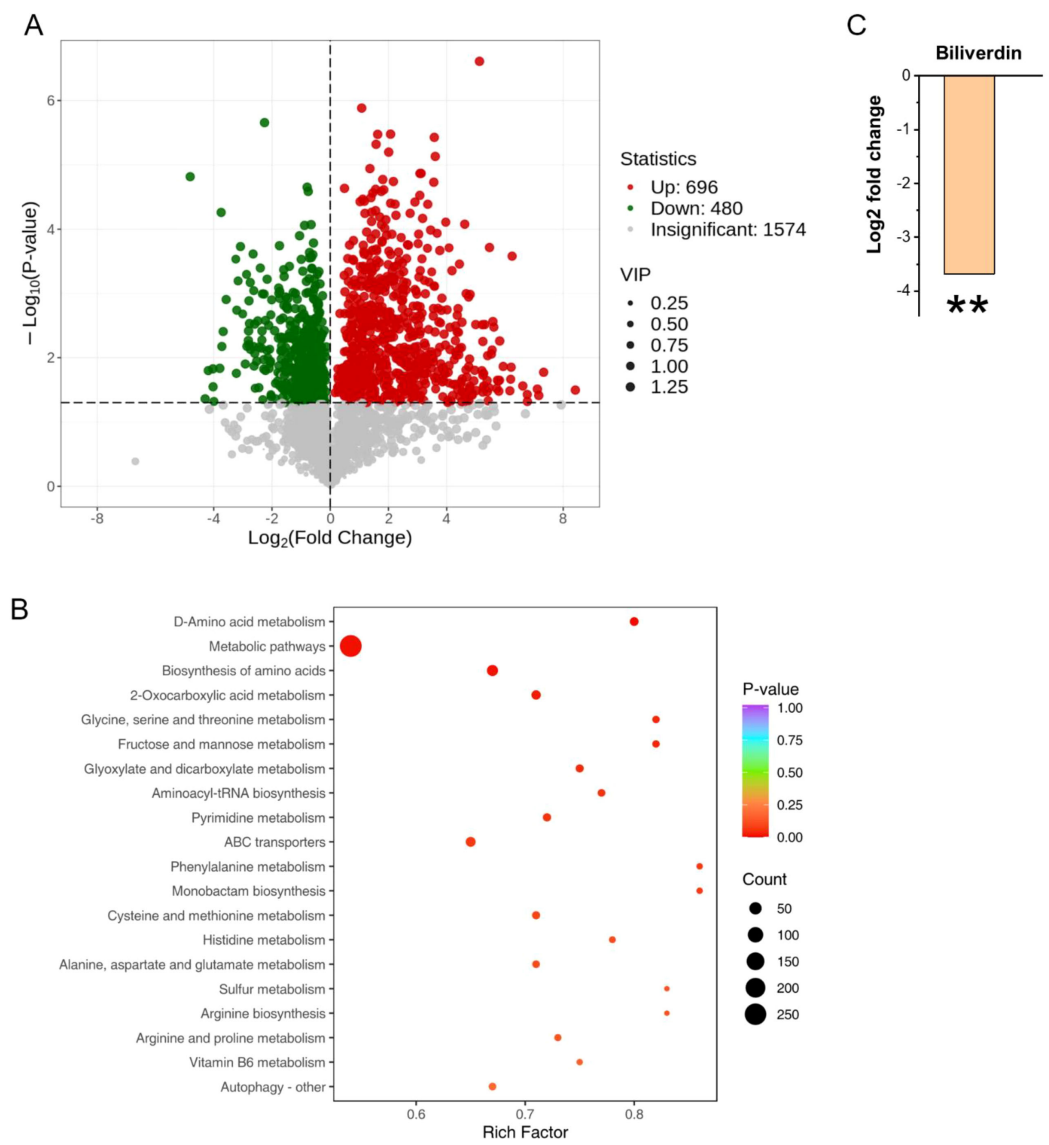
The role of Bud32 in regulating intracellular metabolite levels. **(A)** Loss of *BUD32* gene resulted in 696 up-regulated metabolites (in red) and 480 down-regulated metabolites (in green). **(B)** Enrichment analysis of KEGG pathways showing the top 20 enriched pathways based on differentially expressed metabolites. **(C)** In the case of biliverdin, which is mainly converted from heme, the *BUD32* gene deletion led to a significant decrease in biliverdin levels. ** (Student’s t test, *P*<0.01). Three biological replicates were performed.

Proteomic and metabolomic analyses reveal that the absence of *BUD32* gene leads to significant alterations in both protein and metabolite levels associated with iron homeostasis and metabolism. Specifically, the deletion of *BUD32* resulted in the upregulation of 520 proteins and the downregulation of 291 proteins (**Figure 2A**). This notable shift in the proteomic landscape underscores Bud32 possible role in maintaining the balance of proteins involved in iron ion binding and Fe-S cluster binding (**Figure 2B**, **Supplementary Figure S2** and **S3**). The differentially expressed proteins related to iron transport, iron-sulfur cluster transporters, iron-sulfur cluster (ISC) assembly, and ISC-containing proteins are detailed in **Supplementary Table S2**. Additionally, Gene Ontology (GO) analysis also indicated a downregulation of the KEOPS complex in the cell component category and a decrease in tRNA modification in the biological process category (**Supplementary Figure S2**). The loss of *BUD32* gene profoundly affected intracellular metabolite levels, resulting in the upregulation of 696 metabolites and the downregulation of 480 metabolites (**Figure 3A**). Enrichment analysis of KEGG pathways indicated that the deletion of *BUD32* disrupted metabolic processes (**Figure 3B**). One striking finding is the significant decrease in biliverdin levels in absence of *BUD32* gene (**Figure 3C**). Biliverdin, primarily derived from heme breakdown, serves various functions, including acting as an antioxidant and a signaling molecule. The reduction in biliverdin levels suggested that Bud32 may be involved in the regulation of heme metabolism, possibly by influencing the enzymes responsible for heme catabolism or the availability of substrates and cofactors necessary for this process. Overall, proteomic and metabolomic analyses show that Bud32 is involved in regulating the expression of proteins and metabolites related to iron binding, Fe-S cluster assembly, or heme metabolism. The observed changes underscore the potential role of Bud32 in maintaining both iron homeostasis and metabolic functions.

### 3.4 Bud32 likely modulates Fe-S cluster assembly to influence Grx4, Cir1, and HapX activities

**Figure 4:**
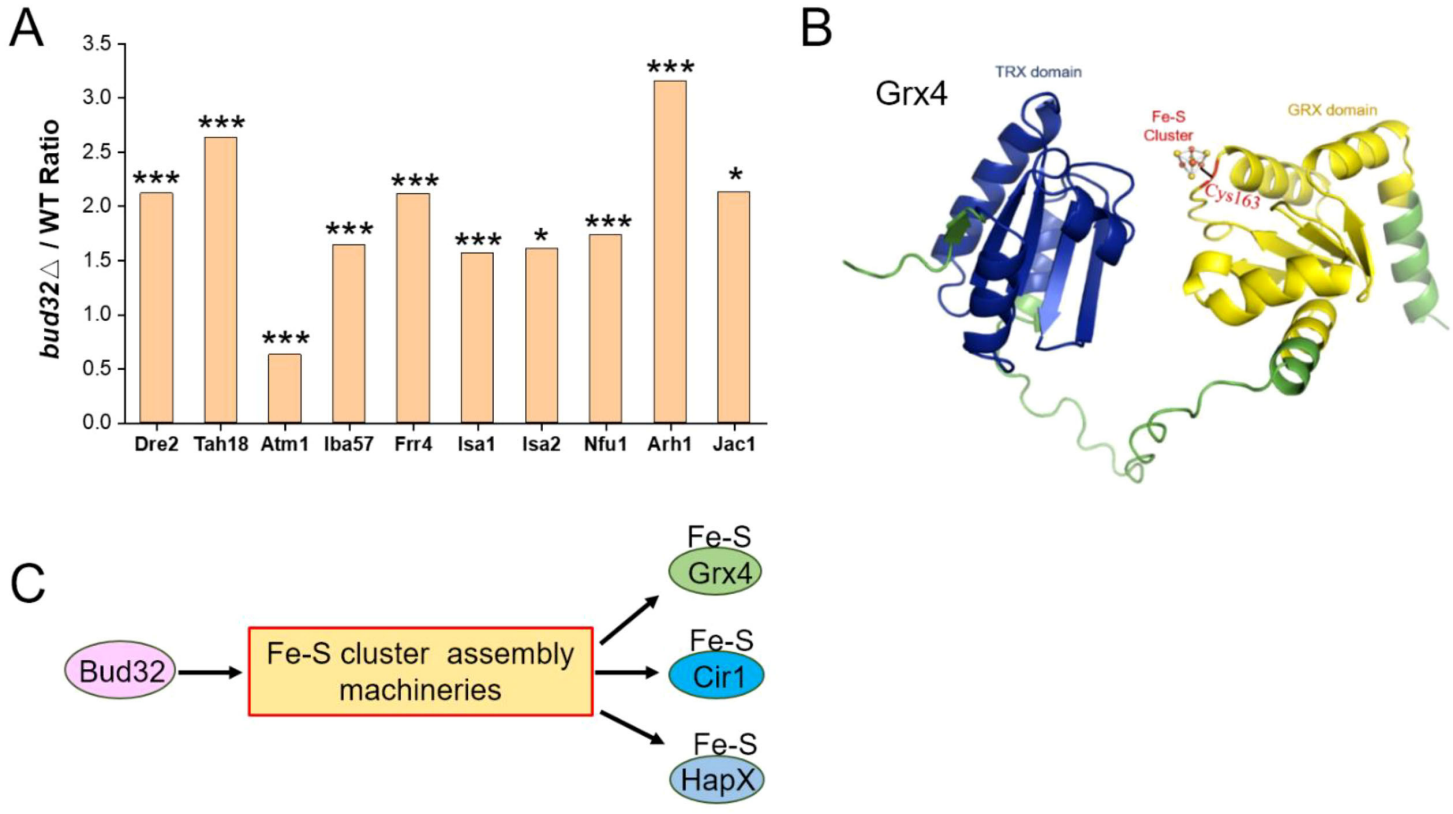
The regulation of Bud32 on the assembly of the Fe-S cluster. **(A)** *BUD32* gene deletion significantly resulted in the expression of proteins involved in the Fe-S cluster assembly machineries, specifically including Dre2, Tah18, Atm1, Iba57, Frr4, Isa1, Isa2, Nfu1, Arh1, and Jac1. The statistical significance of these findings was assessed using a Student’s t-test, with significance levels indicated as follows: * (*P*<0.05), ** (*P*<0.01), and *** (*P*<0.001). Three biological replicates were performed. **(B)** The 3D structural modeling of Grx4 from *Cneoformans* was performed using AlphaFold. The TRX domain and GRX domain of the Grx4 protein are represented in blue and yellow, respectively. Cys163 is highlighted as a potential Fe-S binding site. **(C)** A model proposing that Bud32 probably influences the activities of Grx4, Cir1, and HapX through Fe-S assembly machineries.

Atm1 is involved in iron metabolism and virulence in *C. neoformans*, facilitating the transport of glutathione-coordinated iron-sulfur clusters to the cytosol (61, 62). Notably, we observed a modest reduction in the protein levels of the mitochondrial transporter Atm1 in the *bud32*Δ mutant (**Figure 4A**). The cysteine (Cys) residue within the CGFS motif of CGFS-type monothiol glutaredoxins, including Grx4, is thought to be integral for Fe-S cluster binding (**Figure 4B**) (63–65). Both Cir1 and HapX are known to be Fe-S cluster binding proteins (21, 47). The deletion of *BUD32* gene impacts Fe-S cluster assembly, as indicated by proteomic analyses (**Figure 4C**). Specifically, *BUD32* gene deletion led to altered expression of proteins associated with the Fe-S cluster assembly machinery, including Dre2, Tah18, Atm1, Iba57, Frr4, Isa1, Isa2, Nfu1, Arh1, and Jac1 (**Figure 4A**). Overall, these findings suggest that Bud32 potentially influences the activities of Grx4, Cir1, and HapX through the modulation of Fe-S cluster assembly machinery.

### 3.5 Bud32 regulates the phosphorylation of Cir1 and Rim101

**Figure 5:**
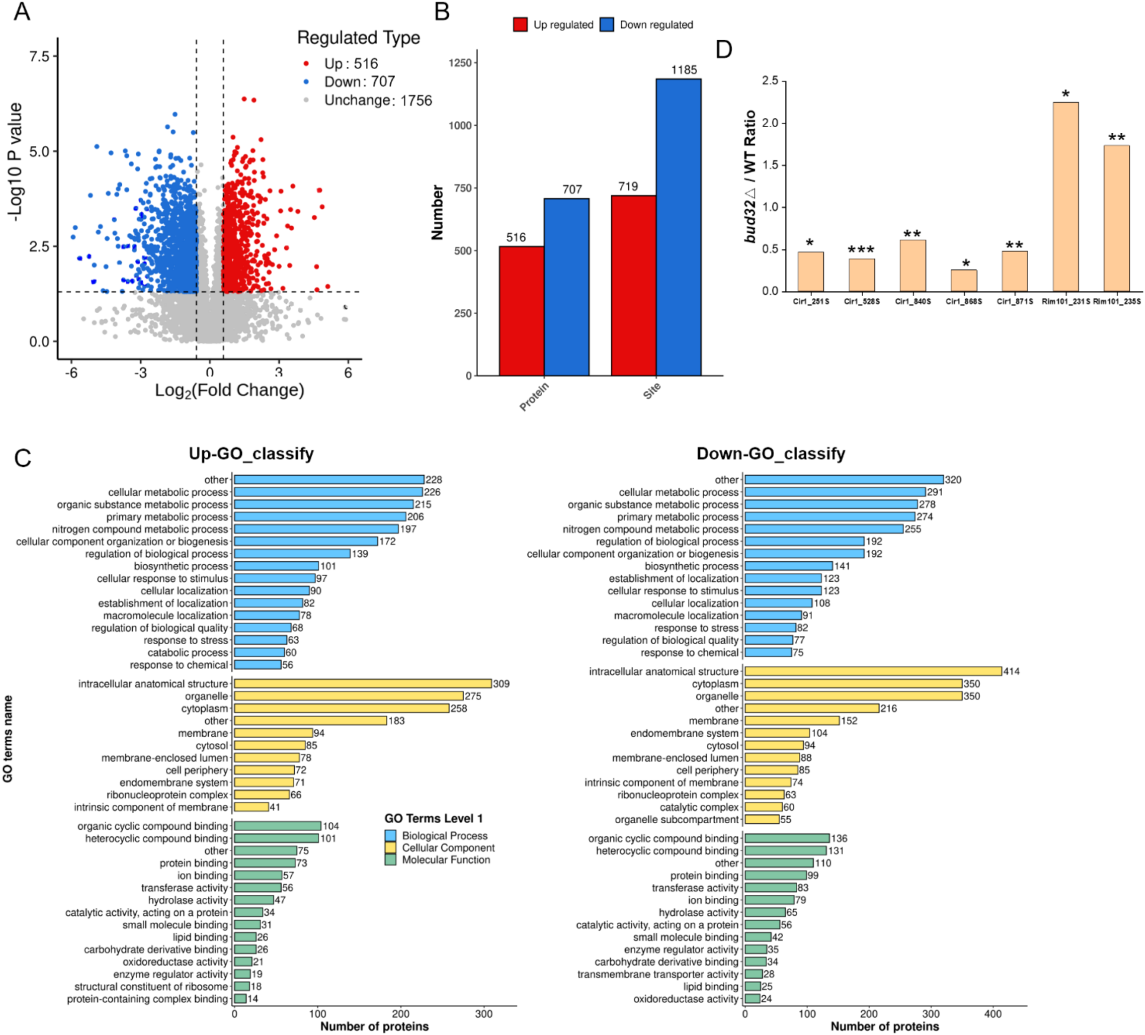
The effects of Bud32 on phosphorylation levels. **(A,B)** Deletion of *BUD32* gene led to up-regulated phosphorylation of 516 proteins (in red) and down-regulated phosphorylation of 707 proteins (in blue). Additionally, loss of *BUD32* gene resulted in 719 up-regulated phosphorylation sites (in red) and 1185 down-regulated phosphorylation sites (in blue). **(C)** GO categories for the differentially phosphorylated proteins. **(D)** *BUD32* deletion influenced the phosphorylation levels of iron regulatory factors Cir1 and Rim101. The phosphorylated Ser sites of Cir1 protein are Ser251, Ser528, Ser840, and Ser868. The phosphorylated Ser sites of Rim101 protein are Ser231 and Ser235. Significance levels are indicated as follows: * (Student’s t test, *P*<0.05), ** (Student’s t test, *P*<0.01), *** (Student’s t test, *P*<0.001), based on three biological replicates.

We next investigated the role of Bud32 in protein phosphorylation. *In vivo* phosphoproteomics analysis revealed that Bud32 was a critical regulator of phosphorylation, specifically influencing the phosphorylation levels of key iron regulatory proteins Cir1 and Rim101 (**Figure 5**). Deletion of *BUD32* gene induced substantial alterations in the phosphorylation status of numerous proteins, suggesting that Bud32 acts as a regulatory element within cellular signaling pathways that govern phosphorylation dynamics. Notably, the absence of Bud32 led to an up-regulation of phosphorylation in 516 proteins and a down-regulation in 707 proteins (**Figure 5A**). This significant shift underscores the importance of Bud32 in maintaining phosphorylation balance within the cell. Furthermore, we observed that 719 phosphorylation sites were up-regulated, while 1185 sites were down-regulated following *BUD32* gene deletion (**Figure 5B**). The extensive number of affected proteins and phosphorylation sites implies that Bud32 may exert broad effects on cellular functions, potentially influencing multiple signaling pathways. To provide deeper insights, we conducted GO analysis of differentially phosphorylated proteins, as illustrated in **Figure 5C**. This analysis enables the identification of biological processes and molecular functions affected by the phosphorylation changes associated with Bud32. Understanding these GO categories will enhance our comprehension of the functional implications arising from *BUD32* gene loss and the potential pathways that may be disrupted in its absence. Additionally, **Figure 5D** highlights the specific case of iron regulatory factors Cir1 and Rim101, both of which exhibit altered phosphorylation levels in the absence of *BUD32* gene. Cir1 possesses several phosphorylated serine residues, including Ser251, Ser528, Ser840, and Ser868, while Rim101 has two critical phosphorylated serine sites, Ser231 and Ser235. Overall, the findings show that Bud32 significantly impacts the phosphorylation status of numerous proteins, including vital iron regulators such as Cir1 and Ri 101.

## 4 Discussion

This study provides new insights into the role of Bud32 in regulating iron homeostasis in *C. neoformans*. Previous research has identified Bud32 as essential for the synthesis of critical virulence factors, including capsule and melanin production (51, 52). Our findings expand upon this knowledge by demonstrating Bud32 significant involvement in iron regulation, revealing an additional dimension of its regulatory role.

Our investigation indicates that the deletion of the *BUD32* gene leads to significant changes in the levels of proteins and metabolites involved in iron homeostasis. We analyzed differentially expressed proteins and identified key disruptions in those associated with iron transport, Fe-S cluster transporters, Fe-S cluster assembly, and ISC-containing proteins. These findings underscore the potential role of Bud32 in maintaining cellular iron homeostasis; its deletion results in the misregulation of these proteins. Such misregulation can severely disrupt essential cellular functions and ultimately impact virulence. Moreover, our metabolomic analysis shows a notable decrease in biliverdin levels following the deletion of *BUD32*. Given that biliverdin is a compound primarily derived from heme, this finding suggests that Bud32 may play a pivotal role in heme metabolism. Understanding the relationship between Bud32 and iron metabolism is particularly relevant for pathogenic fungi such as *C. neoformans*, which must efficiently scavenge iron in the iron-limited environments typical of mammalian hosts. The ability of *C. neoformans* to acquire and utilize iron effectively is critical to its virulence, especially under conditions of limited iron availability. This relationship between Bud32, iron metabolism, and virulence presents important implications for future research on fungal pathogenesis.

Grx4 interacts with the iron regulator Cir1 and is important for maintaining iron homeostasis, as well as being essential for the virulence of *C. neoformans* (45). In *Saccharomyces cerevisiae*, Bud32 is implicated in the phosphorylation of Ser134 on Grx4, which is regulated by the Sch9 kinase (66). Our results indicate that, in *C. neoformans*, while the phosphorylation of Grx4 remains unchanged, key iron regulators Cir1 and Rim101 exhibit significant changes in their phosphorylation status in the absence of *BUD32*. This finding suggests that Bud32 may play a role in the phosphorylation dynamics of Cir1 and Rim101, impacting iron homeostasis and cellular stress responses. Furthermore, the KEOPS complex, which includes Bud32, is involved in various cellular processes, including tRNA modification and transcriptional regulation (51). Our GO analyses confirm the downregulation of the KEOPS complex and related tRNA modification processes in the *bud32*Δ mutant. This suggests that the observed phosphorylation changes in Cir1 and Rim101 may be secondary effects stemming from altered tRNA modification. Our research provides evidence for the involvement of Bud32 in the complex regulation of iron homeostasis in *C. neoformans*. Future studies should aim to elucidate the precise molecular mechanisms by which Bud32 regulates iron homeostasis and their implications for the pathogenicity of *C. neoformans*.

The function of Bud32 and its associated downstream signaling pathways in fungal pathogens like *Aspergillus fumigatus* and *Fusarium graminearum* is not well understood. In *A. fumigatus*, the Bud32 ortholog PipA is essential, but its specific role remains unclear (67). In *F. graminearum*, the Bud32 ortholog FgBud32 is critical for growth, branching, and virulence (68). However, the mechanistic roles of Bud32 in these pathogens have yet to be explored. In conclusion, this study underscores the role of Bud32 in regulating iron homeostasis and virulence factors in *C. neoformans*. It remains to be determined whether the reduced melanin production observed in the *bud32*Δ mutant is due to an intrinsic impairment in melanin biosynthesis or is a secondary effect of its decreased growth rate. The absence of *BUD32* leads to significant disruptions in iron metabolism, affecting proteins involved in iron transport and heme metabolism. Additionally, Bud32 influences the phosphorylation of key iron regulatory proteins, highlighting its importance in signaling pathways related to iron homeostasis.

## Data Availability

Mass spectrometry proteomics data can be accessed through the ProteomeXchange Consortium, specifically under dataset PXD056845 for 4D-FastDIA quantitative proteomics and PXD056847 for phosphoproteomics, hosted in the PRIDE repository. Furthermore, the metabolomics data has been stored in the EMBL-EBI metaboLights database with the identifier MTBLS11462.

## Supplementary material

Supplementary Figure 1 The impact of Bud32 on the three major virulence factors. **(A)** The deletion of *BUD32* gene led to decreased capsule formation. WT strain and three independent *bud32*Δ mutants (*bud32*△#1, *bud32*△#2, *bud32*△#3) were grown in defined low-iron medium at 30°C for 16 h, and capsule formation was assessed by India ink staining for the indicated strains. Scale bar = 5 μm. **(B)** Quantitative analysis of capsule size. For each strain depicted in panel a, fifty cells were measured to evaluate cell diameter, capsule thickness, and the ratio of capsule thickness to cell diameter. The bars presented reflect the average values obtained from these measurements, along with their respective standard deviations. Statistical significance compared to the WT capsule size is marked by **** (ANOVA analysis, *P*<0.0001). **(C)** The *BUD32* gene deletion resulted in the decreased melanin production. Spot assays were performed on L-DOPA plates to assess melanin production for each strain, demonstrating a clear difference in pigment accumulation. **(D)** Temperature sensitivity was assessed for each strain through spot assays on YPD medium at 30°C and 37°C, revealing notable differences in growth patterns.

Supplementary Figure 2 The dotplot of GO analysis for up-regulated proteins or down-regulated proteins in the comparison between WT control and *bud32*Δ mutant. BP, biological process; CC, cell component; MF, molecular function.

Supplementary Figure 3 The GO classify of up-regulated proteins or down-regulated proteins between WT control and *bud32*Δ mutant.

Supplementary Table 1 Lists the primer sequences utilized for the *bud32*Δ mutant in the WT strain. F, forward primer; R, reverse primer.

Supplementary Table 2 Differentially expressed proteins of iron transport/iron-sulfur cluster transporter/iron-sulfur cluster (ISC) assembly/ISC-containing proteins in *bud32*Δ mutant. Significant differences were observed when comparing the *bud32*Δ mutant to the WT control, with fold changes exceeding 1.5 or falling below 0.67, accompanied by a *p*-value of less than 0.05.
