# Supplementary material for: The kinase Bud32 regulates iron homeostasis in fungal pathogen Cryptococcus neoformans
Source: Front Immunol. 2025 Jul 25;16:1624237. doi: 10.3389/fimmu.2025.1624237 (PMC12332752; doi:10.3389/fimmu.2025.1624237)
Supplement: Supplementary Figure 1 — The impact of Bud32 on the three major virulence factors. (A) The deletion of BUD32 gene led to decreased capsule formation. WT strain and three independent bud32Δ mutants (bud32△#1, bud32△#2, bud32△#3) were grown in defined low-iron medium at 30°C for 16 h, and capsule formation was assessed by India ink staining for the indicated strains. Scale bar = 5 μm. (B) Quantitative analysis of capsule size. For each strain depicted in panel a, fifty cells were measured to evaluate cell diameter, capsule thickness, and the ratio of capsule thickness to cell diameter. The bars presented reflect the average values obtained from these measurements, along with their respective standard deviations. Statistical significance compared to the WT capsule size is marked by **** (ANOVA analysis, P<0.0001). (C) The BUD32 gene deletion resulted in the decreased melanin production. Spot assays were performed on L-DOPA plates to assess melanin production for each strain, demonstrating a clear difference in pigment accumulation. (D) Temperature sensitivity was assessed for each strain through spot assays on YPD medium at 30°C and 37°C, revealing notable differences in growth patterns. [file DataSheet1.docx]

**Supplementary Figures and Tables**


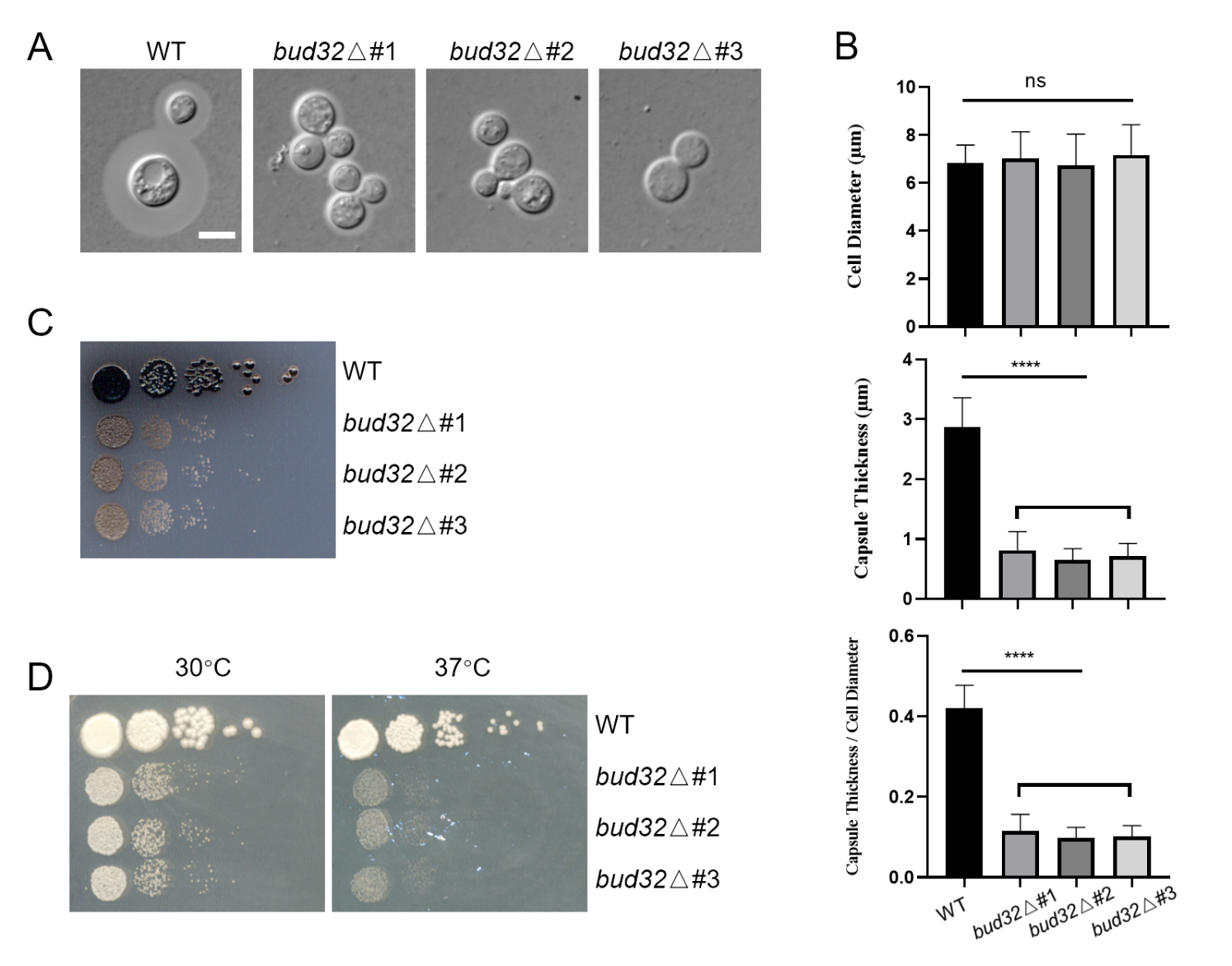


**FIGURE S1**

The impact of Bud32 on the three major virulence factors. **(A)** The deletion of *BUD32* gene led to decreased capsule formation. WT strain and three independent *bud32*Δ mutants (*bud32*△#1, *bud32*△#2, *bud32*△#3) were grown in defined low-iron medium at 30°C for 16 h, and capsule formation was assessed by India ink staining for the indicated strains. Scale bar = 5 μm. **(B)** Quantitative analysis of capsule size. For each strain depicted in panel a, fifty cells were measured to evaluate cell diameter, capsule thickness, and the ratio of capsule thickness to cell diameter. The bars presented reflect the average values obtained from these measurements, along with their respective standard deviations. Statistical significance compared to the WT capsule size is marked by **** (ANOVA analysis, *P*<0.0001). **(C)** The *BUD32* gene deletion resulted in the decreased melanin production. Spot assays were performed on L-DOPA plates to assess melanin production for each strain, demonstrating a clear difference in pigment accumulation. **(D)** Temperature sensitivity was assessed for each strain through spot assays on YPD medium at 30°C and 37°C, revealing notable differences in growth patterns.

**
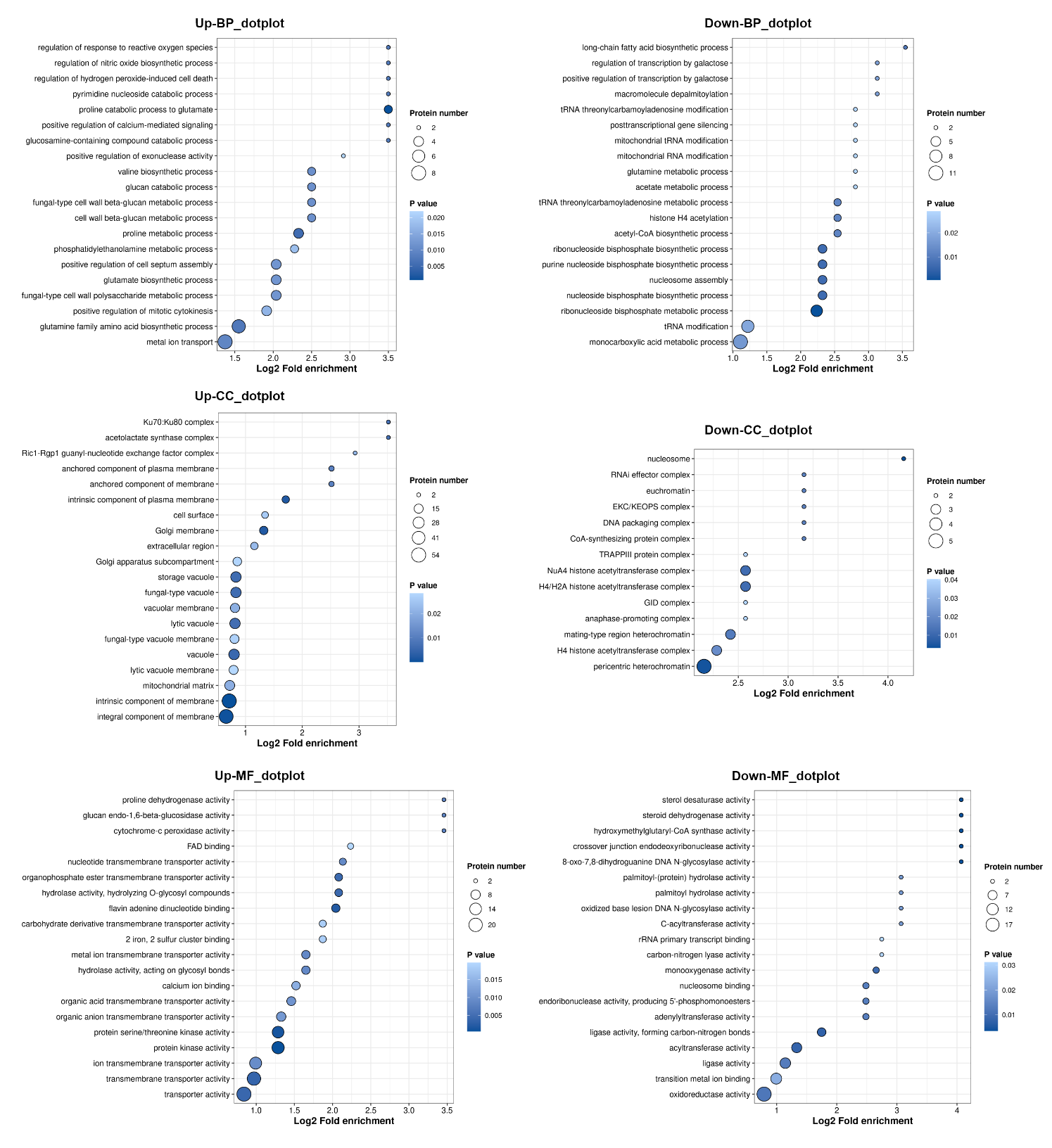
**

**FIGURE S2**

The dotplot of GO analysis for up-regulated proteins or down-regulated proteins in the comparison between WT control and *bud32*Δ mutant. BP: biological process; CC: cell component; MF: molecular function.

**
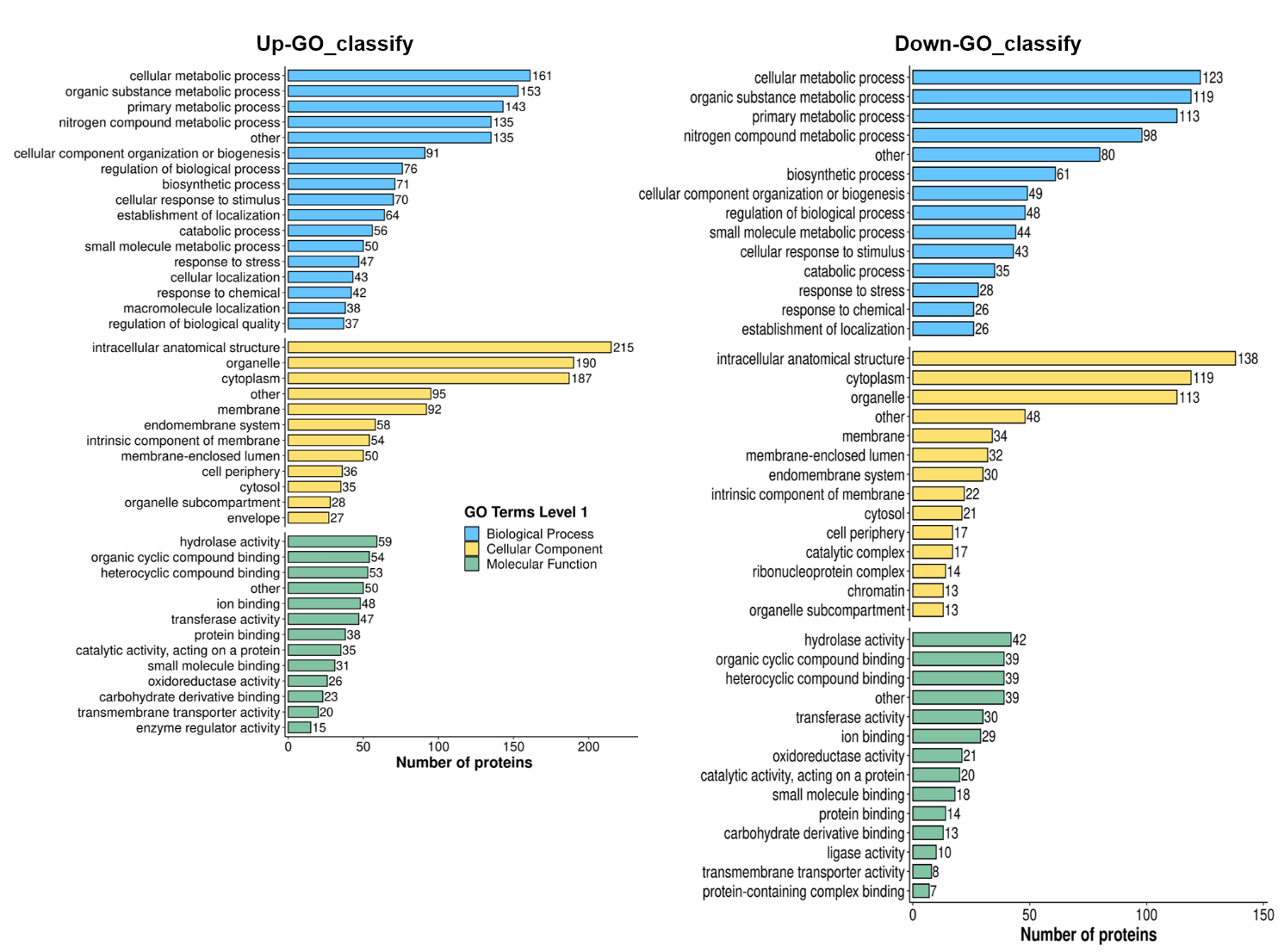
**

**FIGURE S3**

The GO classify of up-regulated proteins or down-regulated proteins between WT control and *bud32*Δ mutant.

**TABLE S1** Lists the primer sequences utilized for the *bud32*Δ mutant in the WT strain. F: forward primer; R: reverse primer.

| **Name** | | **Sequence 5’-3’** | **Reference** | |
| --- | --- | --- | --- | --- |
| 1_ Bud32 up_F | gagatgtcaaagttatggccgat | | | This Study |
| 3_ Bud32 up_R | agctcacatcctcgcagcatgccaaagaccagtgaacaatc | | | This Study |
| 2_ Bud32 (nourseothricin-resistance gene NAT)_F | gattgttcactggtctttggcatgctgcgaggatgtgagct | | | This Study |
| 5_ Bud32 (nourseothricin-resistance gene NAT)_R | acatgcaaaattaccaagtctccagaagagatgtagaaacta | | | This Study |
| 4_ Bud32 down_F | tagtttctacatctcttctggagacttggtaattttgcatgt | | | This Study |
| 6_ Bud32 down_R | cgaaggaagaaagacaagacgaa | | | This Study |
| 7_NE. Bud32_F | tacttctcagcgccttacttttg | | | This Study |
| 8_NE. Bud32_R | ttctttaacgctttcagaccctc | | | This Study |
| 9_PO.Bud32_F | gagatgtcaaagttatggccgat | | | This Study |
| 10_PO.Bud32_R | ccacacaattgaacgaaggaaga | | | This Study |

**TABLE S2** Differentially expressed proteins of iron transport/iron-sulfur cluster transporter/iron-sulfur cluster (ISC) assembly/ISC-containing proteins in *bud32*Δ mutant. Significant differences were observed when comparing the *bud32*Δ mutant to the WT control, with fold changes exceeding 1.5 or falling below 0.67, accompanied by a *p*-value of less than 0.05.

| **Protein** | **Function** | **Ratio (WT vs *bud32*Δ)** |
| --- | --- | --- |
| **Iron transport** | | |
| CNAG_00876 (Fre7) | Ferric-chelate reductase | 0.54 |
| CNAG_05154 (Ccc1) | Membrane fraction protein | 1.77 |
| CNAG_06242 (Cft1) | High-affinity iron permease CaFTR1 | 0.52 |
| **Iron-sulfur cluster transporter** | | |
| CNAG_04358 (Atm1) | Iron-sulfur cluster transporter | 0.63 |
| **Mitochondrial ISC assembly** | | |
| CNAG_00389 (Iba57） | Mitochondrial protein | 1.64 |
| CNAG_05011（Frr4) | ferroxidase | 2.11 |
| CNAG_02131 (Isa1) | Iron-sulfur cluster assembly protein (Fragment) | 1.56 |
| CNAG_00491 (Isa2) | FeS cluster biogenesis domain-containing protein | 1.61 |
| CNAG_03395 (Nfu1) | NifU-like protein c | 1.73 |
| CNAG_05035 (Arh1) | NADPH:adrenodoxin oxidoreductase, mitochondrial | 3.16 |
| CNAG_04288 (Jac1) | Fe-S protein assembly co-chaperone HscB | 2.13 |
| **Cytosolic ISC assembly** | | |
| CNAG_01802 (Dre2) | Fe-S cluster assembly protein | 2.12 |
| CNAG_01839 (Tah18) | NADPH-dependent diflavin oxidoreductase 1 | 2.63 |
| **ISC-containing proteins** | | |
| CNAG_06621 (Bio2) | biotin synthase | 0.64 |
| CNAG_00462 (Cir2) | Electron transfer flavoprotein-ubiquinone oxidoreductase | 1.78 |
